# Novel and Practical Scoring Systems for the Diagnosis of Thyroid Nodules

**DOI:** 10.1371/journal.pone.0163039

**Published:** 2016-09-21

**Authors:** Ying Wei, Xinrong Zhou, Siyue Liu, Hong Wang, Limin Liu, Renze Liu, Jinsong Kang, Kai Hong, Daowen Wang, Gang Yuan

**Affiliations:** 1 Department of Internal Medicine, Tongji Hospital, Huazhong University of Science and Technology, Wuhan, Hubei, China; 2 Molecular Diagnostic Laboratory, Tongji Hospital, Huazhong University of Science and Technology, Wuhan, Hubei, China; 3 Department of surgical cytology, Tongji Hospital, Huazhong University of Science and Technology, Wuhan, Hubei, China; 4 Department of ultrasonic, Tongji Hospital, Huazhong University of Science and Technology, Wuhan, Hubei, China; Universita degli Studi di Napoli Federico II, ITALY

## Abstract

**Objective:**

The clinical management of patients with thyroid nodules that are biopsied by fine-needle aspiration cytology and yield indeterminate results remains unsettled. The *BRAF V600E* mutation has dubious diagnostic value due to its low sensitivity. Novel strategies are urgently needed to distinguish thyroid malignancies from thyroid nodules.

**Design:**

This prospective study included 504 thyroid nodules diagnosed by ultrasonography from 468 patients, and fine-needle aspiration cytology was performed under ultrasound guidance. Cytology and molecular analysis, including *BRAF V600E*, *RET/PTC1* and *RET/PTC3*, were conducted simultaneously. The cytology, ultrasonography results, and mutational status were gathered and analyzed together. Predictive scoring systems were designed using a combination of diagnostic parameters for ultrasonography, cytology and genetic analysis. The utility of the scoring systems was analyzed and compared to detection using the individual methods alone or combined.

**Result:**

The sensitivity of scoring system^a^ (ultrasonography, cytology, *BRAF V600E*, *RET/PTC*) was nearly identical to that of scoring system^b^ (ultrasonography, cytology, *BRAF V600E*); these were 91.0% and 90.2%, respectively. These sensitivities were significantly higher than those obtained using FNAC, genetic analysis and US alone or combined; their sensitivities were 63.9%, 70.7% and 87.2%, respectively. Scoring system^c^ (ultrasonography, cytology) was slightly inferior to the former two scoring systems but still had relatively high sensitivity and specificity (80.5% and 95.1%, respectively), which were significantly superior to those of single cytology, ultrasonography or genetic analysis. In nodules with uncertainty cytology, scoring system^a^, scoring system^b^ and scoring system^c^ could elevate the malignancy detection rates to 69.7%, 69.7% and 63.6%, respectively.

**Conclusion:**

These three scoring systems were quick for clinicians to master and could provide quantified information to predict the probability of malignant nodules. Scoring system^b^ is recommended for improving the detection rate among nodules of uncertain cytology.

## Introduction

In recent years, the incidence of thyroid cancer has increased rapidly worldwide, which may be attributed to the widespread use of high-resolution ultrasonography (US) [1, 2]. Of all incidentally detected thyroid nodules, nearly 90% are benign (BN) and can be managed conservatively [2]. Only a small portion of nodules are malignant and should be surgically resected. Although fine-needle aspiration cytology (FNAC) is the most reliable process to biopsy thyroid nodules, 10% to 40% of all FNAC samples yield indeterminate results [3, 4]. In recent years, with the rapid development of molecular biology, various molecular mutations and arrangements and immunohistochemical markers have emerged [5–9]. Among these, the greatest attention has been given to the BRAF V600E mutation, which is a highly specific molecular marker for papillary thyroid carcinoma (PTC) and has dubious diagnostic value due to its low sensitivity[10, 11]. These detection methods all have limitations and make it difficult to distinguish between benign and malignant tumors. Due to the indeterminacy of diagnosis, many patients have suffered unnecessary interventions and excessive medical treatment, which could be avoided with accurate diagnosis. Therefore, distinguishing accurately between benign nodules and malignant tumors to ensure that each patient receives timely and appropriate treatment is of great concern. Thus, novel strategies are urgently needed to distinguish thyroid malignancies from thyroid nodules.

To overcome and supplement the individual shortcomings of ultrasonography (US), FNAC and *BRAF* mutation analysis, previous studies have combined them in an attempt to predict the probability of PTC[11–14]. Pompili, G. developed an ultrasound-based malignancy score to manage thyroid follicular proliferation, which only included patients in the US [15]. Rago, T. proposed a clinical risk score using FNAC and US to manage patients with indeterminate thyroid nodules, and this score had relatively inferior sensitivity and specificity[16]. Kim, S.K. recently combined US, FNAC and *BRAF V600E* and designed a prediction table and a nomogram as tools to diagnose PTC[11]. The prediction model mentioned in the study provided personalized and quantified information regarding the probability of PTC and emphasized the significance of preoperative *BRAF* mutation analysis in indeterminate thyroid nodules. However, the prediction table could not predict the probability of malignancy, and the nomogram was inconvenient to use. A small control group and few benign nodules limited the study. These studies suffered from a lack of quantified information, inconvenience and inferior sensitivity, which impacted their clinical application. The aim of this study was to design a quantitative and convenient scoring system as a clinical tool to preoperatively estimate the probability of malignancy using a combination of US, FNAB, and molecular markers analysis that can help clinicians and patients easily assess indeterminate nodules preoperatively.

## Methods

### Patient selection

Four hundred sixty-eight patients with at least 1 suspicious thyroid nodule diagnosed by ultrasonography (US) on physical examination in Tongji Hospital from 16 May 2014 to 1 November 2015 were included. Suspicious thyroid nodules were those with at least one of the following suspicious US features: solidity, hypoechogenicity or marked hypoechogenicity, microlobulated to irregularmargin, microcalcifications, and taller-than-wide shape [17, 18]. Each patient’s highest-risk thyroid nodule on US was selected for analysis; if some nodules showed similar characteristics on US, the largest nodule was targeted for biopsy. The research about molecular diagnosis of thyroid nodules has been approved by Tongji Hospital, Huazhong University of Science and Technology. FNAC was performed under ultrasound guidance on these nodules of 468 patients (504 thyroid nodules) who provided written informed consent. For minors (participants <18 years old), their parents signed the informed consent.

### Ultrasonograph

The primary assessment of thyroid nodules was performed using US scanners (HDI 5000 or IU22; Philips Medical Systems, Bothell, WA, USA) equipped with a commercially available 7 to 12-MHz linear-array transducer. US images were interpreted by 1 experienced radiologist for nodule size, shape, internal material, echogenicity, margin characteristics, calcifications, hardness and vascularity. US-detectable thyroid nodules were categorized into the following four patterns according to thyroid imaging reporting and data system (TIRADS): 4a-one suspicious US feature, 4b-two suspicious US features, 4c-three or four suspicious US features, and 5-five suspicious US features [17, 18]. Suspicious US features were mentioned previously.

### Fine-needle aspiration cytology and cytological analysis

The highest-risk thyroid nodules from all patients underwent US-guided FNAC by an experienced sonographer who specialized in thyroid US and a senior technician of the surgical cell chamber who specialized in thyroid puncture. Patients provided written informed consent. US-guided FNAC was performed with a 22-gauge needle attached to a 5-mL disposable plastic syringe. Cell smears were first collected, and the residual cells in syringe were collected in cell lysis buffer. Conventional cytology techniques were used, and air-dried smears were stained with Baso Liu's stain which was similar with romanowsky stain technique. All of the thyroid FNA samples (FNAs) received at the Department of Pathology and Cytology in Tongji Hospital were reported by two cytologists based on the Bethesda System with some minor modification in UK terminology[19, 20]. Cytology results are generally divided into the following categories: Thy1 ‘non-diagnostic for cytological diagnosis’ is broadly equivalent to Bethesda class 1 ‘non-diagnostic or unsatisfactory’, Thy 2 ‘non-neoplastic’ is broadly equivalent to Bethesda class II ‘benign’, Thy 3a ‘neoplasm possible/atypia/non-diagnostic’ is broadly equivalent to Bethesda class III ‘atypia of undetermined significance or follicular lesion of undetermined significance’, Thy 3f ‘neoplasm possible, suggestive of a follicular neoplasm’ is broadly equivalent to Bethesda class IV ‘follicular neoplasm or suspicious for a follicular neoplasm’, Thy 4 ‘suspicious of malignancy’ is broadly equivalent to Bethesda class V ‘suspicious for malignancy’ and Thy 5 ‘malignant’ is broadly equivalent to Bethesda class VI ‘malignant’[19].

### Genetic analysis including *BRAF V600E*, *RET/PTC1* and *RET/PTC3*

Genetic analysis was performed at the Molecular Diagnostics Laboratory of Tongji Hospital, Wuhan, China. First, DNA and RNA were extracted immediately from the cell lysis buffer with a DNA/RNA co-extracting kit (Tiangen biotech, Beijing, China, DP121221) according to the protocol of the manufacturer. Then, the extracted RNA was immediately transcribed using a reverse transcription PCR kit (Tiangen biotech, Beijing, China, KR106). The concentrations of isolated DNA and RNA were measured using a NanoDrop spectrophotometer. The average DNA concentration for the FNAC smears was 21.4 ng/μL(range 5.0–158 ng/μL). Mutation analysis was performed for the *BRAF V600E* using allelic specific primer PCR (ASP-PCR). PCR primer sequences were designed to amplify a 201-bp fragment and the primers were displayed in Table 1. Amplified fragments were separated on agarose gel and visualized by golden view staining. Positive results were then verified through sanger-sequencing. *RET/PTC1* and *RET/PTC3* were detected by real-time PCR with primers designed to flank the respective fusion point on ABI 7500 (Applied Biosystems). The respective primers and probes for *RET/PTC1* and *RET/PTC3* were as following in Table 1. GAPDH was used as the reference gene to evaluate the quality of RNA and the primers were also displayed in Table 1. We could judge the presence of rearrangements according to the fluorescent dissolution curve. The curve will be straight if no rearrangements are present, and it will rise if rearrangements exist.

**Table 1:** The primers or probes for *BRAF*, *RET/PTC1* and *RET/PTC3*.

	Gene	Primers and probes	size
***BRAF***	**wild-F**	**GGTGATTTTGGTCTAGCTAGAGT**	**23**
	**mutant-F**	**GGTGATTTTGGTCTAGCTAGAGA**	**23**
	**common-R**	**TGTGAATACTGGGAACTATGAAAAT**	**25**
***RET/PTC1***	***CCDC6*-F**	**GGAGACCTACAAACTGAAGTGCAA**	**24**
	***CCDC6-RET*-R**	**CCCTTCTCCTAGAGTTTTTCCAAGA**	**25**
	RET/PTC1*	**ACCGCGACCTGCG**	**13**
***RET/PTC3***	***ELE1*-F**	**CCAGTGGTTATCAAGCTCCTTACA**	**24**
	***ELE1-RET*-R**	**GGGAATTCCCACTTTGGATCCTC**	**23**
	RET/PTC3*	**ACCCAGCACCGACC**	**14**
***GAPDH***	***GAPDH*-F**	**CCCCGGTTTCTATAAATTGAGCC**	**23**
	***GAPDH*-R**	**AGCATCGCCCCACTTGATTT**	**20**

*Gene with this sign means the sequence of probes.

### Histopathological results

Surgical specimens were microscopically examined by experienced pathologists and assessed for the following factors: the main pathological type, tumor size (measured the longest diameter of the largest lesion), location, multifocality, extrathyroidal extension, lymphovascular invasion, margin involvement, lymph node metastasis, and underlying thyroid condition such as chronic lymphocytic thyroiditis (CLT).

### Establishment of the scoring system

We aimed to establish a scoring system to predict benign or malignant nodules concisely and conveniently. Six categories of cytology, 4 categories of US and 3 categories of molecular analysis were incorporated into the assessment criteria. Statistical analysis was performed with SPSS version 21.0 software (IBM, Chicago, IL, USA), and P values<0.05 were defined as statistically significant. Binary logistic regression analysis was used to calculate regression coefficients of the diagnostic parameters for the above categories. The diagnostic parameters of US, FNAC, and genetic analysis were assigned scores by their regression coefficient for malignancy using the round-off principle. The receiver-operating characteristic (ROC) curve was analyzed to identify the optimal cut-off value of the scores for the diagnosis of malignancy.

## Results

### Clinicopathological characteristics

In total, of the 468 patients who participated during the study period, 348 were females and had a mean age of 44.8±11.5 years (range 11–77 years), and 120 were males and had a mean age of 45.3±12.1 years (range 15–73 years). The patients with nodules diagnosed as Thy4-5 by FNAC, with positive *BRAF V600E* mutation or *RET/PTC* rearrangement or with more than 3 suspicious US features were suggested to undergo total thyroidectomy and bilateral neck dissection. Other patients with indeterminate FNAC or US results underwent tubercle resection at the suggestion of their clinicians. In summary, 504 nodules were included in our study; 133 nodules were ultimately proven to be malignant, and 371 nodules were proven to be benign.

As shown in Table 2, the cytological results are displayed according to the Bethesda System with some minor modification. For the cytological diagnosis of 504 FNAs from 468 patients, 24 were reported as Thy 5, 62 as Thy 4, 5 as Thy 3f, 32 as Thy 3a, 185 as Thy 2 and 196 as Thy 1. Ultimately, malignancy was found according to pathological analysis in 8.2%(16/196) of the Thy1 nodules, 8.1%(15/185) of the Thy2 nodules, 50%(16/32) of the Thy3a nodules, 20%(1/5) of the Thy3f nodules, 98.4%(61/62) of the Thy4 nodules and 100%(24/24) of the Thy5 nodules. Further, ultrasound findings were collected and classified according to the TIRADS classification system. In the 504 nodules from these patients, 60 nodules were classified as 4a, 164 as 4b, 231 as 4c and 49 as 5. Ultimately, 1.7% (1/60) of the 4a nodules, 9.8% (16/164) of the 4b nodules, 33.8% (78/231) of the 4c nodules and 77.6% (38/49) of the 5 nodules were proven to be malignant after pathological analysis. All FNAs were tested for the *BRAF V600E* mutation and *RET/PTC* rearrangement. Among all nodules, 19.8% (100/504) showed evidence of mutation or rearrangement, whereas 404 nodules had negative molecular analysis. Among the 100 nodules, 18 in Thy 5, 48 in Thy 4, 10 in Thy 3a, 15 in Thy 2 and 5 in Thy 1 were all with *BRAF V600E* mutation; 2 in Thy 2 and 1 in Thy 4 were with *RET/PTC3* arrangement; 1 nodule in Thy 3a was with *RET/PTC1* arrangement. As shown in Table 2, 90 nodules with *BRAF V600E*, 1 nodule with *RET/PTC*1, 3 nodules with *RET/PTC*3 and 39 nodules with negative variations were proven to be malignant after pathological analysis. However, 6 nodules with positive *BRAF V600E* mutation were turned out to be benign in postoperative pathological results. Two cases were classified as 4c in US and Thy3a in FNA, which were turned out to be partial adenomatous hyperplasia with nodular goiter. Two cases with *BRAF V600E* were classified as 4b in US and Thy2 in FNA, which were diagnosed as adenomatous hyperplasia with colloid goiter. One case with 4a in US and Thy2 in FNA was also partial adenomatous hyperplasia with nodular goiter. One case with 4c in US and Thy2 in FNA was diagnosed as partial adenomatous hyperplasia with underlying lymphocytic thyroiditis. Above consequence seemed to break the agreement: *BRAFV600E* mutation only existed in malignant thyroid cancers” [21–24]. However, similar results to ours had been reported in other study[25, 26]. It may imply that molecular variations were the early events in the process of tumorigenesis.

**Table 2:** Clinicopathological characteristics of 504 thyroid nodules, including US, FNAC and genetic analysis.

FNAC	total	BN	MN	US	Total	BN	MN	Molecular analysis	Total	BN	MN
**Thy1**	**196**	**180**	**16**	**4a**	**60**	**59**	**1**	**Negative**	**404**	**365**	**39**
**Thy2**	**185**	**170**	**15**	**4b**	**164**	**148**	**16**	***BRAF V600E***	**96**	**6**	**90**
**Thy3a**	**32**	**16**	**16**	**4c**	**231**	**153**	**78**	***RET/PTC1***	**1**	**0**	**1**
**Thy3f**	**5**	**4**	**1**	**5**	**49**	**11**	**38**	***RET/PTC3***	**3**	**0**	**3**
**Thy4**	**62**	**1**	**61**	**/**				**/**			
**Thy5**	**24**	**0**	**24**	**/**				**/**			
**total**	**504**	**371**	**133**	**total**	**504**	**371**	**133**	**total**	**504**	**371**	**133**

NO, non-operation; BN, benign nodules; MN, malignant nodules; Thy1, non-diagnostic; Thy2, non-neoplastic or benign; Thy3a, neoplasm possible/atypia/non-diagnostic; Thy3f, neoplasm possible suggestive of follicular neoplasm; Thy4, suspicious of malignant; Thy5, malignant; 4a, one suspicious US feature; 4b, two suspicious US features; 4c, three or four suspicious US features; 5, five suspicious US features.

### The three scoring systems

S1 Table shows the 3 scoring systems and their corresponding regression coefficients, which were all statistically significant with P values of approximately 0.000. Scoring system^a^ was the comprehensive scoring system and included US, FNAC, *BRAF V600E* mutation and *RET/PTC* arrangements. Scoring system^b^ included US, FNAC and *BRAF V600E* mutation and excluded *RET/PTC* rearrangements. Scoring system^c^ included only US and FNAC, and this might be suitable for basic hospitals.

All the assigned points of each parameter for every scoring system were based on their regression coefficient and awarded rounded value. The concrete numerical values were shown in Table 3.

**Table 3:** The values of each parameters for every scoring system.

	US				FNAC						Genetic analysis	
	4a	4b	4c	5	Thy2	Thy1	Thy3a	Thy3f	Thy4	Thy5	-	+
**Scoring system**^a^	**0**	**1**	**4**	**6**	**0**	**1**	**2**	**4**	**8**	**6**	**0**	**5**
**Scoring system**^b^	**0**	**1**	**4**	**6**	**0**	**1**	**2**	**4**	**8**	**5**	**0**	**5**
**Scoring system**^c^	**0**	**1**	**4**	**6**	**0**	**0**	**2**	**3**	**8**	**6**	**/**	**/**

^a^ The score system included US, FNAC and genetic analysis including *BRAF V600E* mutation and *RET/PTC* arrangements.

^b^ The score system only included US, FNAC and *BRAF V600E* mutation except *RET/PTC* rearrangements.

^c^ The score system only included US and FNAC, which was suitable for basic hospital.

The ROC curve is shown in S2 Table. The cut-off values were chosen based on the largest youden index and were 6.5 points, 6.5 points and 5 points for scoring system^a^, scoring system^b^ and scoring system^c^, respectively.

### Application of FNAC, US, genetic analysis and the scoring systems

The distribution of postoperative pathological results was shown in Table 4. Due to the high malignancy rates in the cytology of Thy4-5 nodules (98.4% and 100%, respectively) and in the US of 4c-5nodules (33.8% and 81%, respectively), we defined cytology of Thy4-5 as positive cytology and US of 4c-5 as positive US (Table 5). Because of relatively lower malignancy rates in Thy1-3f (8.2%, 8.1%, 50% and 20%, respectively) and in US of 4a-4b (1.7% and 9.8%, respectively), we defined Thy1-3f as negative cytology and US of 4a-4b as negative US. Diagnostic values included sensitivity, specificity, positive predictive value (PPV), negative predictive value (NPV), false negative rate (FNR), false positive rate (FPR) and youden index. In 133 malignant nodules, the sensitivities of FNAC, US and genetic analysis were 63.9% (85/133), 87.2% (116/133) and 70.7% (94/133), respectively. The sensitivities of scoring system^a^ and scoring system^b^ using cut-off values of 6.5 points were nearly identical and were 91.0% (121/133) and 90.2% (120/133), respectively. These were significantly higher than FNAC, US or genetic analysis alone. We also combined FNAC-US-genetics (FUg) together for analysis, as shown in Table 5. FUg-A, FUg-B and FUg-C respectively denoted if either one, either two or all three of FNAC-US-genetics were positive, then it would be considered as positive. With the highest sensitivity but the lowest specificity of Fug-A and with the highest specificity but the lowest sensitivity of Fug-C, only FUg-B has medium sensitivity and specificity (78.9% and 99.2%, respectively) but with the highest youden index. However, comparing with scoring system^a^ and scoring system^b^, FUg-B was obviously inferior due to the lower sensitivity and youden index. It demonstrated that these two scoring systems had the best authenticity based on the youden index. US alone had relatively higher sensitivity (87.2%) but lower specificity (55.8%) and could be used for simple screening of large populations. Together, the above data demonstrated that scoring system^a^ and scoring system^b^ using cut-off values of 6.5 points substantially increased the sensitivity and decreased the rate of missed diagnosis. To cater to basic-level hospitals, scoring system^c^ was developed without genetic analysis, and its sensitivity, NPV and youden index were slightly inferior to the former two scoring systems but still significantly superior to genetic analysis and FNAC alone. Nevertheless, due to its similar diagnostic value and lower expense, scoring system^b^ is recommended as the most practical scoring system.

**Table 4:** The distribution of postoperative pathological results.

	FNAC		Genetic analysis		US analysis		FUg-A		FUg -B		FUg -C		Score system^a^		Score system^b^		Score system^c^	
	+	-	+	-	+	-	+	-	+	-	+	-	≥6.5	<6.5	≥6.5	<6.5	≥5	<5
**Total(n = 504)**	**86**	**418**	**100**	**404**	**280**	**224**	**300**	**204**	**108**	**396**	**58**	**446**	**131**	**373**	**130**	**374**	**125**	**379**
**PTC(n = 130)**	**83**	**47**	**92**	**38**	**114**	**16**	**129**	**1**	**103**	**27**	**57**	**73**	**119**	**11**	**118**	**12**	**105**	**25**
**FTC(n = 1)**	**0**	**1**	**0**	**1**	**1**	**0**	**1**	**0**	**0**	**1**	**0**	**1**	**0**	**1**	**0**	**1**	**0**	**1**
**FvPTC(n = 2)**	**2**	**0**	**2**	**0**	**1**	**1**	**2**	**0**	**2**	**0**	**1**	**1**	**2**	**0**	**2**	**0**	**2**	**0**
**TM(n = 133)**	**85**	**48**	**94**	**39**	**116**	**17**	**132**	**1**	**105**	**28**	**58**	**75**	**121**	**12**	**120**	**13**	**107**	**26**
**BN(n = 371)**	**1**	**370**	**6**	**365**	**164**	**207**	**168**	**203**	**3**	**368**	**0**	**371**	**10**	**361**	**10**	**361**	**18**	**353**

+, positive; -, negative; TM, total malignancy; BN, benign.

FUg-A, Combining FNAC, US and genetics to analyze, either one of the 3 methods was positive, then as positive; FUg-B, Combining FNAC, US and genetics to analyze, either two of the 3 methods were positive, then as positive; FUg-C, Combining FNAC, US and genetics to analyze, only all of the 3 methods were positive, then as positive.

^a^ The score system included US, FNAC and genetic analysis including *BRAF V600E* mutation and *RET/PTC* arrangements.

^b^ The score system only included US, FNAC and *BRAF V600E* mutation except *RET/PTC* rearrangements.

^c^ The score system only included US and FNAC, which was suitable for basic hospital.

**Table 5:** Application of FNAC, US, Genetic analysis and score system in thyroid cancers.

	FNAC	Genetic analysis	US analysis	FUg-A	FUg -B	FUg -C	Score system^a^	Score system^b^	Score system^c^
**sensibility**	**63.9%**	**70.7%**	**87.2%**	**99.2%**	**78.9%**	**43.6%**	**91.0%**	**90.2%**	**80.5%**
**specificity**	**99.7%**	**98.4%**	**55.8%**	**54.7%**	**99.2%**	**100%**	**97.3%**	**97.3%**	**95.1%**
**FPR**	**0.3%**	**1.6%**	**44.2%**	**45.3%**	**0.8%**	**0%**	**2.7%**	**2.7%**	**4.9%**
**FNR**	**36.1%**	**29.3%**	**12.8%**	**0.7%**	**21.1%**	**56.4%**	**9.0%**	**9.8%**	**19.5%**
**PPV**	**98.8%**	**94.0%**	**41.4%**	**44%**	**97.2%**	**100%**	**92.4%**	**92.3%**	**85.6%**
**NPV**	**88.5%**	**90.3%**	**92.4%**	**99.5%**	**92.9%**	**83.2%**	**96.8%**	**96.5%**	**93.1%**
**Youden index**	**0.636**	**0.691**	**0.430**	**0.539**	**0.790**	**0.436**	**0.883**	**0.875**	**0.756**

FNR, false negative rate; FPR, false positive rate; PPV, positive predictive value; NPV, negative predictive value.

FUg-A, Combining FNAC, US and genetics to analyze, either one of the 3 methods was positive, then as positive; FUg-B, Combining FNAC, US and genetics to analyze, either two of the 3 methods were positive, then as positive; FUg-C, Combining FNAC, US and genetics to analyze, only all of the 3 methods were positive, then as positive.

^a^ The score system included US, FNAC and genetic analysis including *BRAF V600E* mutation and *RET/PTC* arrangements.

^b^ The score system only included US, FNAC and *BRAF V600E* mutation except *RET/PTC* rearrangements.

^c^ The score system only included US and FNAC, which was suitable for basic hospital.

To estimate the predicted probability of malignant nodules, 233 uncertain nodules including non-diagnostic Thy1 and indeterminate Thy3a-3f on FNAC were reevaluated with these scoring systems. The Thy4 category of indeterminate nodules should be included in the above analysis, but it was excluded due to the high diagnostic rate of malignancy of approximately 98.4% (61/62). As shown in Table 6, after surgery, 33 nodules were shown to be malignant, and 200 nodules were benign. Among the 33 malignant nodules, 42.4% (14/33) had positive genetic analysis. Meanwhile, scoring system^a^, scoring system^b^ and scoring system^c^ could help to elevate the detection rates to 69.7% (23/33), 69.7% (23/33) and 63.6% (21/33), respectively. In the benign nodules, both genetic analysis and the scoring systems had high detection rates.

**Table 6:** The application of three score systems in 233 uncertain nodules.

Indeterminacy (n = 233)	Genetic analysis	Score system^a^	Score system^b^	Score system^c^
**Malignant(n = 33)**	**Negative (n = 19)**	**<6.5 points (n = 10)**	**<6.5 points (n = 10)**	**<5.0 points (n = 12)**
	**Positive (n = 14)**	**>6.5 points (n = 23)**	**>6.5 points (n = 23)**	**>5.0 points (n = 21)**
**Elevated detection rate**	**42.4%**	**69.7%**	**69.7%**	**63.6%**
**Benign (n = 200)**	**Negative(n = 198)**	**<6.5 points (n = 192)**	**<6.5 points (n = 192)**	**<5.0 points (n = 189)**
	**Positive (n = 2)**	**>6.5 points (n = 8)**	**>6.5 points (n = 8)**	**>5.0 points (n = 11)**
**Elevated detection rate**	**99.0%**	**96.0%**	**96.0%**	**94.5%**

^a^ The score system included US, FNAC and genetic analysis including *BRAF V600E* mutation and *RET/PTC* arrangements.

^b^ The score system only included US, FNAC and *BRAF V600E* mutation except *RET/PTC* rearrangements.

^c^ The score system only included US and FNAC, which was suitable for basic hospital.

## Discussion

This simple perspective study established three quantitative and convenient scoring systems as clinical tools to preoperatively estimate the malignancy of thyroid nodules with a combination of US, FNAC and molecular analysis. As mentioned previously, the current well-established detection methods for thyroid cancers are US, FNAC, and molecular analysis, which mainly consists of *BRAF V600E*, but each method has its own shortcomings and insufficiency [3, 4, 10, 11].

In this research, the sensitivity of ultrasound for malignant nodules was the most superior (87.2%) of the common detection means, but it had relatively inferior specificity (55.8%), PPV (41.4%) and high false positive rate (FPR, 44.2%). Yoon, J.H. et.al found the diagnostic performance of TIRADS when considering category 3 as negative and categories 4a to 5 as positive to be as follows: sensitivity, 97.4%; specificity, 29.3%; PPV, 23.3%; NPV,98.1%; and accuracy, 41.6%[18]. Although they used different standards, most studies reached analogous conclusions of higher sensitivity but lower specificity, which also demonstrates that US is an excellent screening method for large populations but not a definite diagnostic tool. On the contrary, FNAC has been established as the main tool with which to identify malignant thyroid nodules and had much higher specificity (99.7%), PPV (98.8%) and lower FPR (0.3%) but relatively inferior sensitivity (63.9%) and a high false negative rate (36.1%), which is similar to prior studies[11, 27]. Its main limitations are non-diagnostic and indeterminate specimens, which accounted for 46.2% (233/504) of all US-guided results in this study; this is slightly higher than that reported (10% to 40%) in previous studies [3, 4, 9]. Among the 233 uncertain nodules, 14.2% (33/233) were found to be malignant after surgery, while up to 26.6% of nodules in a study by Krane, J.F. on an American population and nearly 27.8% of nodules in a study by Agretti, P. on an Italian population were found to be malignant[28, 29]. In addition, of 185 nodules with Thy2 cytology, 8.1% (15/185) were malignant on postoperative pathology. However, malignancy rates of only 1–3% have been reported in large retrospective series that analyzed the utility of systematic repeat FNA in nodules with prior benign cytology results [30–33]. However, these studies were retrospective and used repeated cytology. The malignancy in Thy2 nodules was approximately 6% (2/32) in the study by Krane, J.F, where pathological results were received in only 32/132 Thy2 nodules [28]. Different puncture needles, methods and techniques may have contributed to these differences. Race may be a factor that cannot be ignored. Together, the above data demonstrated that a significant proportion of malignant nodules escaped diagnosis by FNAC.

As is well known, PTC, the most common thyroid carcinoma, frequently carries *BRAF* and *RET/PTC*, which have been identified as highly specific markers of thyroid cancer [34]. Many studies support the feasibility of detecting *BRAF* or *RET/PTC* in thyroid FNA samples and have shown that this may improve cytological FNA diagnosis [35–38]. Because PTC is the most common thyroid carcinoma, we included *BRAF* and *RET/PTC* in our research. In this study, the sensitivity (70.7%), specificity (98.4%), PPV (94.0%), NPV (90.3%), FPR (1.6%) and FNR (29.3%) of genetic analysis were all between the two methods mentioned previously. However, the youden index of genetic analysis was superior. A prospective study of 314 Chinese individuals reported the following diagnostic parameters of *BRAF* and *RET/PTC*: sensitivity (88.7%), specificity (100%), PPV (100%), NPV (91.56%)[39]. This difference may be affected by factors such as age, the techniques used, geographic region, and histological subtype of PTC. However, in 100 nodules with positive variation, only 1 nodule with *RET/PTC1* and 3 nodules with *RET/PTC3* were detected, and the remaining nodules all showed *BRAF V600E* mutation; these account for 0.2%, 0.6%, and 19.0% of all nodules, respectively. Due to fewer *RET/PTC* rearrangements, we developed and analyzed scoring system^b^ exclusive of *RET/PTC* rearrangements. Nevertheless, the limitation is that the variant molecules used in this research nearly always indicated papillary thyroid cancers (PTCs), which were the most common malignancies in this study. Other specific and sensitive molecular markers related to other malignancies were scarce, and increasing the detected molecules was time consuming and costly. The birth of next generation sequencing (NGS) brought a new dawn for the dilemma. NGS could test more molecular targets simultaniously and fastly. However, it was much expensive nearly 3000 RMB (about 460 USD) and spending 3 days while only 800 yuan and 1 day of our currently combining detection. Together, these three methods each have their own shortcomings and insufficiency. Novel strategies are urgently needed to distinguish thyroid malignancies from thyroid nodules.

To overcome and supplement the individual shortcomings of US, FNAC and *BRAF* mutation analysis, previous studies have combined them in an attempt to predict the probability of PTC [11–14]. In a large retrospective study of 3297 patients from South Korean, Kim, S.K. recently designed a prediction table and a nomogram as tools to diagnose PTC using the combined analysis of US, FNAB and *BRAF V600E* mutation. His study provided personalized and quantified information of the probability of PTC and also emphasized the significance of preoperative *BRAF* mutation analysis in indeterminate thyroid nodules [11]. However, the nomogram was not convenient to use for clinical physicians and had no specific boundary. In addition, the study of Kim, S.K. only concentrated on papillary thyroid cancers and included a tiny control group comprising 5.8% of patients; there were very few benign nodules. Rago, T. also established a clinical risk score including FNAC and US to manage patients with indeterminate thyroid nodules in a retrospective evaluation and proposed a formula to calculate the scores and corresponding risk[16]. He stated that the presence of Thy3f cytology, the coexistence of 3 suspicious US features, and age<40 years were significantly associated with cancer. Nevertheless, this clinical score had relatively inferior sensitivity (68.2%) and specificity (53.7%) for the assessment of malignancy. Some scholars may propose that testing multiple variant molecules also could increase the detection efficiency (e.g. Thyroseq NGS). Nikiforov YE. reported that ThyroSeq NGS panel, which simultaneously tests for point mutations in 13 genes and for 42 types of gene fusions that occur in thyroid cancer, performed with 90% sensitivity, 93% specificity, a PPV of 83%, a NPV of 96% and 92% accuracy[40]. However, it was much expensive and time-consuming while comparing with our currently combining detection. And the NGS is not widespread as the cutting-edge technology in China.

In this study, we established three scoring systems with different advantages and usage. The parameters of scoring system^a^ were as follows: sensitivity, 91.0%; specificity, 97.3%; PPV, 92.4%; NPV, 96.8%; FPR, 2.7%; FNR, 9.0%; and youden index, 0.883 (Table 5). Almost identical to scoring system^a^, the parameters of scoring system^b^ with the same cut-off value of 6.5 points were 90.2%, 97.3%, 92.3%, 96.5%, 2.7%, 9.8% and 0.875, respectively. The sensitivities of these two systems were significantly higher than those of FNAC (63.9%), US (87.2%) or genetic analysis (70.7%) alone. While comparing with Fugs, these two systems were in a medium. However, the best youden indexindicated the practical value of the two scoring systems. Between the two systems, scoring system^b^ is recommended as the better practical scoring system due to its similar diagnostic value and lower cost. In addition, as for many basic hospitals, genetic analysis could not be conducted due to poor medical equipment. Thus, we found that scoring system^c^, which excludes genetic analysis, is suitable for basic hospitals. The sensitivity (80.5%), NPV (93.1%) and youden index (0.756) were slightly inferior to the former two scoring systems but still significantly superior to single genetic analysis and FNAC. The above evidence demonstrates the practical value of the scoring systems, which could be used widely by both grass-roots hospitals and large general hospitals.

Furthermore, a great deal of studies have shown that preoperative genetic analysis combined with US and FNAC findings could increase the diagnostic accuracy in indeterminate thyroid nodules, which was confirmed in our study[12, 41–45]. Table 6 demonstrates that genetic analysis identified 42.4% of the nodules with uncertainty cytology and increased the detection rate. Nevertheless, scoring system^a^ and scoring system^b^ helped elevate the detection rate in uncertain nodules to 69.7% with a cut-off value of 6.5 points, and this detection rate was much higher than that of genetic analysis alone. Apart from genetic analysis, scoring system^c^ also helped to elevate the detection rate in uncertain nodules to 63.6% with a cut-off value of 5.0 points. The above data show that the three scoring systems could improve the detection rate in uncertain nodules significantly above genetic analysis alone, which again confirms the practical value of the scoring systems.

Certainly, this study still had several limitations. First, this study was conducted in a Chinese population where *BRAF V600E* was relatively prevalent, and thus these results might not be applicable to other countries. Second, this study had few samples and very few non-PTC primary thyroid cancers. Analysis of additional patients with various types of primary thyroid cancers should be performed to confirm the strength and value of these scoring systems. To our knowledge, this was the first study to propose quantitative and convenient scoring systems that are easy to master and that can predict primary thyroid cancers.

## Conclusions

In conclusion, we strongly recommend the use of the score systems developed in this study, especially scoring system^b^ and scoring system^c^, which are suitable for large general hospitals and grass-roots hospitals, respectively. The scoring systems are quick for clinicians to master and can easily assess the probability of malignancy in the preoperative period. They provide quantified information to predict the probability of malignant nodules in variable situations.

## Supporting Information

S1 Dataset The related minimal dataset about the manuscript. (XLSX)

S1 Fig The electrophoretogram result about the sensitivity of ASP-PCR. From the right to the left, the bands were 100%NPA, 30%, 25%, 20%, 15%, 10%, 5%, 1% mutant DNA and 100%WT in sequence. The corresponding specimens up and down are the same sample.
(DOCX)

S1 Table Binary logistic regression analysis for diagnostic parameters of US, FNAC and mutation analysis. 4a in ultrasonography, Thy2 in cytology and negative genetic analysis were used as a reference.
(DOCX)

S2 Table ROC curve analysis results. The minimum limit value was the smallest observation which minus 1, and the maximum limit value was the largest observation which add 1. All the other limit values were the mean value of two neighboring observations.
(DOCX)
