# Supplementary material for: Novel and Practical Scoring Systems for the Diagnosis of Thyroid Nodules
Source: PLoS One. 2016 Sep 21;11(9):e0163039. doi: 10.1371/journal.pone.0163039 (PMC5031406; doi:10.1371/journal.pone.0163039)
Supplement: S1 Fig — From the right to the left, the bands were 100%NPA, 30%, 25%, 20%, 15%, 10%, 5%, 1% mutant DNA and 100%WT in sequence. The corresponding specimens up and down are the same sample. (DOCX) [file pone.0163039.s002.docx]

S1 Figure The electrophoretogram result about the sensitivity of ASP-PCR.

100%NPA

100%WT

H2O

30%

25%

20%

15%

10%

5%

1%

100%WT

100%NPA

Mutant type

Wild type


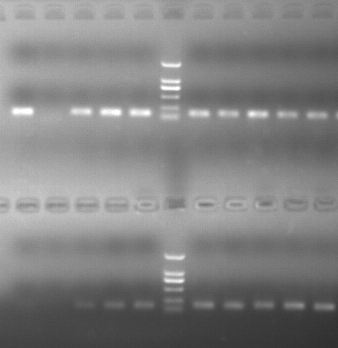


*From the right to the left, the bands were 100%NPA, 30%, 25%, 20%, 15%, 10%, 5%, 1% mutant DNA and 100%WT in sequence. The corresponding specimens up and down are the same sample.
