# Supplementary material for: Novel and Practical Scoring Systems for the Diagnosis of Thyroid Nodules
Source: PLoS One. 2016 Sep 21;11(9):e0163039. doi: 10.1371/journal.pone.0163039 (PMC5031406; doi:10.1371/journal.pone.0163039)
Supplement: S1 Table — 4a in ultrasonography, Thy2 in cytology and negative genetic analysis were used as a reference. (DOCX) [file pone.0163039.s003.docx]

**S1 Table Binary logistic regression analysis for diagnostic parameters of US, FNAB, and mutation analysis.**

| **Score system^a^** | | |  | **Score system^b^** | | |  | **Score system^c^** | | |
| --- | --- | --- | --- | --- | --- | --- | --- | --- | --- | --- |
|  | **B** | **Sig.** |  |  | **B** | **Sig.** |  |  | **B** | **Sig.** |
| **Thy-2** |  | **.000** |  | **Thy-2** |  | **.000** |  | **Thy-2** |  | **.000** |
| **Thy-1** | **1.327** | **.706** |  | **Thy-1** | **.820** | **.135** |  | **Thy-1** | **-.117** | **.770** |
| **Thy-3a** | **2.379** | **.028** |  | **Thy-3a** | **2.266** | **.002** |  | **Thy-3a** | **2.353** | **.000** |
| **Thy-3f** | **4.387** | **.001** |  | **Thy-3f** | **3.808** | **.013** |  | **Thy-3f** | **2.524** | **.084** |
| **Thy-4** | **8.275** | **.000** |  | **Thy-4** | **7.733** | **.000** |  | **Thy-4** | **7.512** | **.000** |
| **Thy-5** | **5.938** | **.039** |  | **Thy-5** | **5.395** | **.000** |  | **Thy-5** | **5.502** | **.000** |
| **US-4a** |  | **.004** |  | **US-4a** |  | **.000** |  | **US-4a** |  | **.000** |
| **US-4b** | **.623** | **.005** |  | **US-4b** | **.665** | **.685** |  | **US-4b** | **1.206** | **.525** |
| **US-4c** | **3.598** | **.000** |  | **US-4c** | **3.660** | **.024** |  | **US-4c** | **4.144** | **.028** |
| **US-5** | **5.804** | **.000** |  | **US-5** | **5.552** | **.001** |  | **US-5** | **5.626** | **.004** |
| **G(-)** |  | **.000** |  | **G(-)** |  | **.000** |  | **constant** | **-5.945** | **.002** |
| **G(+)** | **5.212** | **.000** |  | **G(+)** | **4.589** | **.000** |  |  |  |  |
| **constant** | **-7.286** | **.000** |  | **constant** | **-6.752** | **.000** |  |  |  |  |

**G, genetic analysis; B, regression coefficient.**

**^a^ The score system included US, FNAC and genetic analysis including *BRAF V600E* mutation and *RET/PTC* arrangements.**

**^b^ The score system only included US, FNAC and *BRAF V600E* mutation except *RET/PTC* rearrangements.**

**^c^ The score system only included US and FNAC, which was suitable for basic hospital.**
