# Supplementary material for: Novel and Practical Scoring Systems for the Diagnosis of Thyroid Nodules
Source: PLoS One. 2016 Sep 21;11(9):e0163039. doi: 10.1371/journal.pone.0163039 (PMC5031406; doi:10.1371/journal.pone.0163039)
Supplement: S2 Table — The minimum limit value was the smallest observation which minus 1, and the maximum limit value was the largest observation which add 1. All the other limit values were the mean value of two neighboring observations. (DOCX) [file pone.0163039.s004.docx]

| **Score system^a^** | | | | **Score system^b^** | | | | **Score system^c^** | | | |
| --- | --- | --- | --- | --- | --- | --- | --- | --- | --- | --- | --- |
| **cutoff score** | **sensitivity** | **1 － specificity** | **Youden index** | **cutoff score** | **sensitivity** | **1 － specificity** | **Youden index** | **cutoff score** | **sensitivity** | **1 － specificity** | **Youden index** |
| **-1.000** | **1.000** | **1.000** | **0.000** | **-1.000** | **1.000** | **1.000** | **0.000** | **-1.000** | **1.000** | **1.000** | **0.000** |
| **.500** | **1.000** | **.944** | **0.056** | **.500** | **1.000** | **.944** | **0.056** | **.500** | **1.000** | **.844** | **0.156** |
| **1.500** | **1.000** | **.648** | **0.352** | **1.500** | **1.000** | **.648** | **0.352** | **2.000** | **.992** | **.481** | **0.511** |
| **2.500** | **1.000** | **.489** | **0.511** | **2.000** | **1.000** | **.489** | **0.511** | **3.500** | **.985** | **.452** | **0.533** |
| **3.500** | **1.000** | **.462** | **0.538** | **3.500** | **1.000** | **.462** | **0.538** | ***5.000*** | ***.803*** | ***.051*** | ***0.752*** |
| **4.500** | **.985** | **.285** | **0.700** | **4.500** | **.985** | **.285** | **0.700** | **6.500** | **.682** | **.011** | **0.671** |
| **5.500** | **.924** | **.059** | **0.865** | **5.500** | **.924** | **.059** | **0.865** | **7.500** | **.674** | **.005** | **0.669** |
| ***6.500*** | ***.909*** | ***.030*** | ***0.879*** | ***6.500*** | ***.909*** | ***.027*** | ***0.882*** | **8.500** | **.621** | **.003** | **0.618** |
| **7.500** | **.871** | **.016** | **0.855** | **7.500** | **.871** | **.016** | **0.855** | **9.500** | **.538** | **.000** | **0.538** |
| **8.500** | **.818** | **.011** | **0.807** | **8.500** | **.818** | **.011** | **0.807** | **11.000** | **.439** | **.000** | **0.439** |
| **9.500** | **.705** | **.005** | **0.700** | **9.500** | **.682** | **.005** | **0.677** | **13.000** | **.129** | **.000** | **0.129** |
| **10.500** | **.667** | **.005** | **0.662** | **10.500** | **.667** | **.005** | **0.662** | **15.000** | **.000** | **.000** | **0.000** |
| **11.500** | **.606** | **.000** | **0.606** | **11.500** | **.583** | **.000** | **0.583** |  |  |  |  |
| **12.500** | **.530** | **.000** | **0.530** | **12.500** | **.530** | **.000** | **0.530** |  |  |  |  |
| **13.500** | **.523** | **.000** | **0.523** | **13.500** | **.523** | **.000** | **0.523** |  |  |  |  |
| **14.500** | **.439** | **.000** | **0.439** | **15.000** | **.364** | **.000** | **0.364** |  |  |  |  |
| **16.000** | **.364** | **.000** | **0.364** | **16.500** | **.311** | **.000** | **0.311** |  |  |  |  |
| **18.000** | **.098** | **.000** | **0.098** | **18.000** | **.098** | **.000** | **0.098** |  |  |  |  |
| **20.000** | **.000** | **.000** | **0.000** | **20.000** | **.000** | **.000** | **0.000** |  |  |  |  |

**S2 Table ROC curve analysis results.**

The minimum limit value was the smallest observation which minus 1, and the maximum limit value was the largest observation which add 1. All the other limit values were the mean value of two neighboring observations.
